# A risk scoring system to predict the risk of new‐onset hypertension among patients with type 2 diabetes

**DOI:** 10.1111/jch.14322

**Published:** 2021-07-12

**Authors:** Cheng‐Chieh Lin, Chia‐Ing Li, Chiu‐Shong Liu, Chih‐Hsueh Lin, Mu‐Cyun Wang, Shing‐Yu Yang, Tsai‐Chung Li

**Affiliations:** ^1^ School of Medicine College of Medicine China Medical University Taichung Taiwan; ^2^ Department of Family Medicine China Medical University Hospital Taichung Taiwan; ^3^ Department of Medical Research China Medical University Hospital Taichung Taiwan; ^4^ Department of Public Health College of Public Health China Medical University Taichung Taiwan; ^5^ Department of Healthcare Administration College of Medical and Health Science Asia University Taichung Taiwan

**Keywords:** hypertension, risk scoring system, type 2 diabetes

## Abstract

Hypertension (HTN), which frequently co‐exists with diabetes mellitus, is the leading major cause of cardiovascular disease and death globally. This study aimed to develop and validate a risk scoring system considering the effects of glycemic and blood pressure (BP) variabilities to predict HTN incidence in patients with type 2 diabetes. This research is a retrospective cohort study that included 3416 patients with type 2 diabetes without HTN and who were enrolled in a managed care program in 2001–2015. The patients were followed up until April 2016, new‐onset HTN event, or death. HTN was defined as diastolic BP (DBP) ≥ 90 mm Hg, systolic BP (SBP) ≥ 140 mm Hg, or the initiation of antihypertensive medication. Cox proportional hazard regression model was used to develop the risk scoring system for HTN. Of the patients, 1738 experienced new‐onset HTN during an average follow‐up period of 3.40 years. Age, sex, physical activity, body mass index, type of DM treatment, family history of HTN, baseline SBP and DBP, variabilities of fasting plasma glucose, SBP, and DBP and macroalbuminuria were significant variables for the prediction of new‐onset HTN. Using these predictors, the prediction models for 1‐, 3‐, and 5‐year periods demonstrated good discrimination, with AUC values of 0.70–0.76. Our HTN scoring system for patients with type 2 DM, which involves innovative predictors of glycemic and BP variabilities, has good classification accuracy and identifies risk factors available in clinical settings for prevention of the progression to new‐onset HTN.

## 1 INTRODUCTION

The global prevalence of diabetes mellitus (DM) and hypertension (HTN) continually rises, and both have emerged as major medical and public health concerns. According to the 9th edition of the IDF Diabetes Atlas, the projected number of adults living with diabetes will increase from 463 million (9.3%) to 700 million by 2045 (10.9%).^1^ About 35%–70% of diabetes‐associated vascular complications in diabetic population, including cardiovascular diseases, stroke, lower extremity amputations, chronic renal disease, diabetic retinopathy, and blindness, have been attributed to HTN.^2^ In addition, DM and HTN share similar risk factors, such as obesity, dyslipidemia, insulin resistance, and gene,^3^ with HTN showing a significantly higher prevalence in diabetic patients. Compared with the nondiabetic population, the prevalence of HTN is 1.5–2.0 times more common in the diabetic population.^4^ The coexistence of DM and HTN must be avoided to reduce the microvascular and macrovascular complications of diabetes. Risk prediction models for HTN must be developed to lower its incidence and to improve its prevention in population with diabetes.

The benefits of predictive models are the quantification of the strength of associations for measurable and modifiable risk factors and generation of risk estimates. These point systems can be utilized by nurse practitioners, physicians, and health professionals without the need for understanding complex statistical models because the point systems require simple calculation. In addition, clinicians can be guided by these point systems for their decision making regarding treatments and assistance in motivating patients to modify their behaviors. Another strength of this point system is that patients can easily estimate and monitor their disease risks over time. Published prediction models have been developed to predict HTN, primarily in general and patient populations, using electronic health records (EHRs).^5^, ^6^, ^7^, ^8^, ^9^, ^10^, ^11^, ^12^, ^13^, ^14^, ^15^, ^16^, ^17^, ^18^, ^19^, ^20^, ^21^, ^22^, ^23^, ^24^ Although diabetes is a significant predictor of new‐onset HTN,^11^, ^15^, ^25^ no prediction model has been created for patients with type 2 diabetes. The prediction models for HTN risks in general and patient populations cannot consider diabetes‐specific predictors, such as diabetes duration, poor glycemic control, and diabetes medication.^26^ In addition, glycemic and blood pressure (BP) variabilities are novel factors that are associated with diabetic microvascular and macrovascular complications, arousing the interest of researchers in the field. The potential biological mechanisms of these factors arise from oxidative stress,^27^ which induces endothelial injury and thus increases cardiovascular risk.^28^ A new predictive model considering glycemic and BP variabilities, diabetes duration, poor glycemic control, and diabetes medication for HTN in patients with type 2 diabetes must be constructed. Thus, the current study aimed to develop and validate a point system to estimate HTN risks in patients with type 2 diabetes.

## 2 METHODS

### 2.1 Study patients

We conducted a retrospective cohort study among patients who enrolled in the Diabetes Case Management program (DCMP) of China Medical University Hospital (CMUH), a case management program set up by National Health Insurance Administration in 2001. Enrollees comprised patients with a diagnosis of type 2 diabetes (International Classification Disease, Ninth Revision, Clinical Modification (ICD‐9‐CM); Code of 250). All cases enrolled in the registry between November 2001 and April 2015 had to be continuously enrolled in DCMP until April 2016, new‐onset HTN event, or death. Therefore, this cohort is open or dynamic, that is, each patient joined the study at different time points. The rationale for this criterion is that we involved persons who can provide at least 1‐year of follow‐up to depict the glycemic and BP variabilities. We excluded patients with type 1 diabetes (ICD‐9‐CM code 250.×1/×3), gestational diabetes (ICD‐9‐CM code 648.83) (*n* = 448), patients aged under 30 years and more than 85 years (*n* = 655), HTN at baseline (*n* = 13 603), lack of baseline information (*n* = 251), and follow‐up < 1 year (*n* = 669). A total of 2747 enrolled diabetic patients were randomly assigned to the derivation and validation sets in a 2:1 ratio. Figure S1 shows the flowchart for study patient selection. Ethical approval was obtained from the Ethical Review Board of CMUH (CMUH109‐REC2‐166).

### 2.2 Data source

The data source was the computerized database of Taiwanese patients with type 2 diabetes enrolled in the DCMP of a medical center in Taichung, Taiwan. This database provides information of patient with diabetes, including annual self‐care education and assessment, annual eye examinations, and four laboratory tests annually. The laboratory tests include fasting plasma glucose (FPG), Hemoglobin A1C (HbA1c), creatinine, urine albumin–creatinine ratio, total cholesterol (TC), triglyceride (TG), high‐density lipoprotein (HDL), and low‐density lipoprotein (LDL). The DCMP provides a unique opportunity to quantify the overall impact of lifestyle factors, including body mass index (BMI), smoking, physical activity, and alcohol consumption. The medication information includes oral hypoglycemic agents, insulin, cardiovascular medications (eg, calcium channel blockers), lipid‐lowering agents (eg, statins [HMG‐CoA reductase inhibitors]), and kidney disease medication. In addition to laboratory and pharmaceutical data regulated by DCMP program for reimbursement, information about education programs for nursing care, nutrition, diet, physical activity, smoking, and weight control behaviors were collected.

### 2.3 Measurements

Upon enrolment in the DCMP program, the study patients had a series of medical tests for urine, blood, lifestyle behaviors, body measurements, and medical history gathered at baseline and annually through standardized computerized questionnaire administered by a case manager.

#### 2.3.1 Socio‐demographic factors, lifestyle behaviors, and diabetes‐related variables

The socio‐demographic factors comprised age at baseline, sex, and family histories of diabetes, HTN, hyperlipidemia, and obesity. Lifestyle behaviors of smoking, alcohol drinking, and physical activity were each divided into two subgroups: yes versus no. Diabetes‐related variables included duration and early onset of type 2 diabetes, defined as diabetes onset age ≥ 40 years.

#### 2.3.2 Anthropometric measurement

Weight and height were measured with an auto‐anthropometer (super‐view, HW‐666), with the patients shoeless and wearing light clothing. BMI was derived from the formula: weight (kg)/(height)^2^ (m^2^). Individuals were measured BP in the right arm using the suitable size cuff and a standard tunnel type electronic sphygmomanometer (OMRON, HBP‐9020, Japan) in a seated position without distraction. The instructions for taking BP measurements ask individuals to roll up the sleeve on their arm, to rest in a chair next to a table for 5–10 min with arm resting comfortably at heart level, sitting up straight with their back against the chair, legs uncrossed, and to rest their forearm on the table with the palm of their hand facing up. Usually individuals had one BP measurement. If an individual had two or more BP measurements in a day, the average of these BP measurements was recorded. BP measurements used for calculating the variability were those within 1 year of entry to DCMP for those who had at least two BP measurements.

#### 2.3.3 Laboratory examination

Blood was drawn from an antecubital vein in the morning after a 12 h overnight fasting and was sent for analysis within 4 h of blood collection. Biochemical markers, such as FPG, HbA1c, HDL, LDL, TC, TG, and creatinine, were analyzed by a biochemical auto‐analyzer (Beckman Coulter Synchron system, Lx‐20, Fullerton, CA, USA) at the Clinical Laboratory Department of China Medical University Hospital. FPG in the obtained blood was measured using NAF TUBE. NAF TUBE contained 5 mg sodium fluoride to inhibit glucose metabolism and 4 mg potassium oxalate to chelate calcium and prevent coagulation. HbA1c level was measured using a boronate‐affinity high‐performance liquid chromatography assay (reference range: 4.6%–6.5%). TC and TG were measured in serum mode. TG levels were determined by an enzymatic colorimetric method. HDL and LDL levels were measured by a direct method. Urine test consisted of protein urinalysis and 24 h urinary protein excretion. Urinary albumin‐to‐creatinine ratio (ACR) in the morning urine sample was determined by urinary creatinine (Jaffe's kinetic method) and albumin (colorimetyl bromcresol purple), which were measured by an autoanalyzer. Urinary ACR ranging from 30 to 300 mg/g creatinine was defined as microalbuminuria and above 300 mg/g creatinine as macroalbuminuria.

The estimated glomerular filtration rate (eGFR) was estimated based on serum creatinine levels, in accordance with the Chronic Kidney Disease Epidemiology Collaboration equation.^29^ The measurements for calculating glycemic variability were FPG or HbA1c measurements within 1 year of entry to DCMP for those who had at least two measurements.

#### 2.3.4 Medication‐related variables

The variables for pharmacologic agent use were derived from the dataset of DCMP program. The types of anti‐diabetes treatment containing various oral hypoglycemic agents, such as, metformin, sulfonylurea, thiazolidinedione, meglitinide, and biguanide, and insulin therapy were extracted. Other medication‐related variables included kidney disease medications, HTN medications, cardiovascular medications, and lipid‐lowering medications. All these medications were each divided into two categories: yes versus no.

#### 2.3.5 Comorbidities

Baseline comorbidities consisted of hyperlipidemia, coronary artery disease, severe hypoglycemia, postural hypotension, peripheral neuropathy, nephropathy, diabetic ketoacidosis, and hyperglycemic hyperosmolar nonketotic coma. All the comorbidities were each divided into two classes: yes versus no.

#### 2.3.6 Outcome measures

The main outcome measure was HTN event, which was determined by at least two systolic blood pressure (SBP) and diastolic blood pressure (DBP) measurements. The onset of HTN is defined as one of the following two criteria of 2020 International Society of Hypertension Global Hypertension Practice Guidelines:^30^ SBP ≥140 mm Hg or DBP ≥90 mm Hg; individuals on anti‐hypertensive medications. All patients were followed up from the index date until April 2016 or until a new‐onset HTN event, death, or withdrawal from the DCMP.

### 2.4 Statistical analysis

Means with standard deviations (SDs) of continuous variables and proportions of categorical variables were used to describe baseline characteristics of all study patients. The glycemic and BP variabilities were adjusted for the numbers of visit to reduce measurement bias. The CVs of FPG, HbA1c, SBP, and DBP were divided by the square root of the ratio of total visits to total visits minus one.^31^ The standardized effect size was used to compare the differences in baseline characteristics between the derivation and validation sets. Crude and multivariate‐adjusted hazard ratios with 95% confidence intervals (CIs) for risk or protective predictors of HTN were evaluated by Cox proportional hazard models.

The derivation set was used to generate a prediction model, and the validation set was used for assessment of the predictive accuracy. Then, the steps from the Framingham heart study was used as guides to construct the risk score function.^32^ The steps are shown in Supplement A.

The area under curve (AUC) of receiver operating characteristic (AUROC) curve for 1‐, 3‐, and 5‐year of HTN incidence from probabilities of logistic regressions model was applied to assess the predictive accuracy of the HTN risk prediction model. The correct Harrell's C‐statistic of the AUC was also applied to time‐to‐event analysis. The AUC can be used as the index for assessing the capability of the model to correctly discriminate study patients into HTN or non‐HTN cases. The values of AUC ranged from 0 to 1, where a value higher than 0.7 indicates good discriminatory capability of the model. For the assessment of the discriminatory capability of the risk model, we compared three subgroups with low, medium, and high sum risk scores determined by tertiles of the total score in the validation set. Calibration of the HTN risk prediction model for the validation set was tested by Hosmer–Lemeshow $x^{2}$ method. Internal validation was performed to correct the potential for overfitting or “optimism” by using 1000 times of bootstrap resampling.^33^ Model calibration was carried out to assess the agreement between model‐predicted and observed probabilities. Calibration‐in‐large approach was used to calculate the intercept for evaluation of the extent to which predictions are systematically extremely low or extremely high. The value of intercept zero suggests the lack of systematic estimation of predicted probabilities. Furthermore, calibration slope was estimated for the extremeness of predicted probabilities. If the value of slope was close to one, then model overfitting was not observed. The mean absolute error in the calibration for slope and intercept was revealed during calibration assessment; the error indicates the discrepancy between the observed and bias‐corrected calibrated values. The net reclassification improvement (NRI) and integrated discrimination improvement (IDI) values were used to assess the added value of our scoring system compared with USA's HTN risk score.^24^ All statistical analyses were conducted by SAS version 9.4 (SAS Institute Inc., Cary, NC, USA). Significance level was set at two‐tailed *p* < .05.

## 3 RESULTS

The derivation and validation sets comprised 2278 and 1138 patients, respectively. The two sets contained 1162 (derivation set) and 576 (validation set) cases of new‐onset HTN. The mean (SD) duration of follow‐up was 3.40 (2.51) years. Table 1 provides the detailed baseline characteristics of the derivation and validation set. Among the patients, 1208 (53.03%) and 592 (52.02%) were males, with mean age of 56 years and mean BP of 125 (systolic) and 78 mm Hg (diastolic) in the derivation and validation sets, respectively. The values of all standardized effect sizes < 0.1 indicated no differences in the baseline characteristics between the derivation and validation sets.

**TABLE 1:** Baseline characteristics of the entire study population in the derivation and validation sets

	Derivation set (*n* = 2278)	Validation set (*n* = 1138)	
Variables	MEAN±SDor *n* (%)	MEAN±SDor *n* (%)	Standardized effect size
***Socio‐demographic factors***			
Age (years)	55.72±11.35	55.33±11.57	0.03
Male	1208 (53.03)	592 (52.02)	0.02
Education			
0–5 years	330 (14.49)	174 (15.29)	‐0.02
6–12 years	1368 (60.05)	688 (60.46)	‐0.01
≥ 13 years	580 (25.46)	276 (24.25)	0.03
***Lifestyle behaviors***			
Smoking	456 (20.02)	232 (20.39)	‐0.01
Drinking	201 (8.82)	100 (8.79)	0.00
Physical activity	1251 (54.92)	598 (52.55)	0.05
Body mass index (kg/m^2^)	24.75±3.55	24.84±3.56	‐0.03
***Diabetes‐related factors and biomarkers***			
Family history of diabetes	1540 (67.60)	779 (68.45)	‐0.02
Family history of hypertension	554 (24.32)	267 (23.46)	0.02
Family history of hyperlipidemia	158 (6.94)	63 (5.54)	0.06
Family history of obesity	294 (12.91)	154 (13.53)	‐0.02
Duration of type 2 diabetes (years)	4.97±5.96	4.77±6.51	0.03
Type of DM treatment			
Diet‐only	167 (7.33)	100 (8.79)	‐0.05
Any hypoglycemic drug	2111 (92.67)	1038 (91.21)	0.05
Fasting blood glucose (mg/dl)	152.21±39.38	150.86±38.12	0.03
HbA1c level (%)	7.76±1.44	7.71±1.43	0.03
Variation of FPG (%)	18.16±12.27	17.81±13.16	0.03
Variation of HBA1c (%)	7.76±7.00	7.87±7.13	‐0.02
Systolic blood pressure (mm Hg)	125.21±11.28	125.03±11.12	0.02
Diastolic blood pressure (mm Hg)	77.96±7.6	77.57±7.74	0.05
Variation of SBP (%)	6.16±4.01	6.14±4.25	0.00
Variation of DBP (%)	6.86±4.25	6.91±4.46	‐0.01
eGFR (ml/min/1.73 m^2^)	91.39±20.38	91.46±20.37	0.00
Creatinine (mg/dl)	0.86±0.46	0.87±0.57	‐0.02
SGPT (μ/L)	32.67±33.75	30.57±31.51	0.06
Total cholesterol (mg/dl)	190.69±42.59	191.52±42.94	‐0.02
Triglyceride (mg/dl)	155.51±232.4	152.1±153.54	0.02
High‐density lipoprotein (mg/dl)	43.11±12.23	43.78±11.79	‐0.06
Low‐density lipoprotein (mg/dl)	115.81±35.26	115.54±35.76	0.01
***Comorbidities***			
Stroke	41 (1.80)	22 (1.93)	‐0.01
Hyperlipidemia	422 (18.53)	228 (20.04)	‐0.04
Coronary Artery Disease	67 (2.94)	22 (1.93)	0.06
Severe hypoglycemia	29 (1.27)	8 (0.70)	0.06
Postural hypotension	163 (7.16)	73 (6.41)	0.03
Peripheral neuropathy	206 (9.04)	81 (7.12)	0.07
Nephropathy	283 (12.42)	141 (12.39)	0.00
DKA	17 (0.75)	7 (0.62)	0.02
HHNK	24 (1.05)	16 (1.41)	‐0.03
Microalbuminuria	579 (25.42)	277 (24.34)	0.02
Macroalbuminuria	59 (2.59)	36 (3.16)	‐0.03
Previous cardiovascular diseases	510 (22.39)	223 (19.60)	0.07
***Medication use***			
Cardiovascular medication	216 (9.48)	102 (8.96)	0.02
Lipid‐lowering medication	224 (9.83)	106 (9.31)	0.02
Kidney disease medication	4 (0.18)	4 (0.35)	‐0.04
***Outcomes***			
Hypertension	1162 (51.01)	576 (50.62)	0.01

Previous cardiovascular diseases was defined as previous cardiovascular diseases, including stroke, CAD, nephropathy, and neuropathy.

*Abbreviations*: SD, standard deviation; DM, diabetes mellitus; FPG, fasting plasma glucose; SBP, systolic blood pressure; DBP, diastolic blood pressure; eGFR, estimated Glomerular filtration rate; SGPT, serum glutamic‐pyruvic transaminase; DKA, diabetic ketoacidosis; HHNK, hyperglycemic hyperosmolar nonketotic coma.

Tables 2 and 3 show the results from univariate and multivariate Cox models for building up HTN prediction model in the derivation set, respectively. In Table 2, the multivariate Cox model using backward selection procedure revealed that age, education level, physical activity, body mass index, family history of HTN, type of DM treatment, SBP, DBP, FPG‐CV, SBP‐CV, DBP‐CV, and macroalbuminuria were independent predictors of new‐onset HTN. Although sex was not significant, sex was considered in the multivariate model because it has been reported that men have greater increases in prevalence of HTN compared with women since the 1940s^34^ and women at ages 20–44 years had lower incidences of developing HTN compared with their male counterparts.^35^ Based on the above variables, a point‐based risk‐scoring system of HTN with a range of 0–48 was developed for patients with type 2 diabetes. As shown in Table 3, this scoring system was assigned to graded scores based on the β values of 13 predictors. The graded scores were assigned as follows: age of 30–34 years, 0 points; age of 35–39 years, 1 point; age of 40–44 years, 2 point; age of 45–49 years, 3 point, age of 50–54 years, 4 points; age of 55–59 years, 5 points; age of 60–64 years, 6 points; age of 65–69, 7 points; age of 70–74 years, 8 points; age of 75–79 years, 9 points; age of 80–84, 10 points; male sex, 1 point; non‐physical activity, 2 points; BMI of 18.5–24.9 kg/m^2^, 7 points; BMI ≥ 25 kg/m^2^, 8 points; family history of HTN, 2 points; use of hypoglycemic drug, 3 points; SBP of 120–129 mm Hg, 4 points; SBP ≥ 130 mm Hg, 7 points; DBP ≥ 80 mm Hg, 4 points; FPG‐CV of 10.9–20.0%, 1 points; FPG‐CV ≥ 20.1%, 2 points; SBP‐CV of 4.0%–7.1%, 2 points; SBP‐CV ≥ 7.2%, 3 points; DBP‐CV ≥8.1%, 2 points and macroalbuminuria, 4 points. Using the total score from the above variables, a patient's 1‐, 3‐, and 5‐year new‐onset HTN risks were estimated (Table S1). For calculating individual risks in clinical practice, a woman (0 point) aged 58 years (5 points) practiced regular physical activity (0 point), had a BMI of 25 kg/m^2^ (8 points), had a family history of HTN (2 points), was taking oral anti‐diabetes medicine (3 points), had a baseline SBP of 125 mm Hg (4 points), a baseline DBP of 79 mm Hg (0 point), FPG variation of 15% (1 point), SBP variation of 5% (2 points), and DBP variation of 7% (0 points) has a sum of risk points of 25. Her 1‐, 3‐, and 5‐year risks of HTN should be 24.24%, 48.17%, and 63.08%, respectively.

**TABLE 2:** Cox models estimated hazard ratio and 95% confidence intervals of new‐onset hypertension in derivation set

	HR (95% CI)	
Variables	Crude	Adjusted
***Socio‐demographic factors***		
Age (years)	1.02 (1.01, 1.03)***	1.02 (1.02, 1.03)***
Male	1.12 (1.00, 1.25)	1.10 (0.97, 1.23)
***Lifestyle behaviors***		
Physical activity		
No	1.22 (1.09, 1.37)***	1.18 (1.05, 1.33)**
Yes	1.00	1.00
Body mass index (kg/m^2^)		
<18.5	1.00	1.00
18.5–24.9	2.16 (1.25, 3.75)**	1.97 (1.13, 3.42)*
≥25.0	2.98 (1.72, 5.16)***	2.35 (1.35, 4.08)**
***Diabetes‐related factors and biomarkers***		
Family history of hypertension		
No	1.00	1.00
Yes	1.23 (1.07, 1.41)**	1.28 (1.11, 1.48)***
Type of DM treatment		
Diet‐only	1.00	1.00
Any hypoglycemic drug	1.40 (1.11, 1.75)**	1.30 (1.03, 1.64)*
Systolic blood pressure (mm Hg) at baseline		
<120	1.00	1.00
120–129	1.70 (1.45, 1.99)***	1.47 (1.25, 1.74)***
130–139	2.75 (2.36, 3.21)***	2.06 (1.73, 2.46)***
Diastolic blood pressure (mm Hg) at baseline		
<80	1.00	1.00
80–89	1.78 (1.59, 2.00)***	1.47 (1.29, 1.69)***
Variation of FPG (%)		
<10.9	1.00	1.00
10.9–20.0	1.13 (0.98, 1.31)	1.07 (0.92, 1.24)
≥20.1	1.28 (1.11, 1.48)***	1.17 (1.01, 1.35)*
Variation of SBP (%)		
<4.0	1.00	1.00
4.0–7.1	1.20 (1.04, 1.39)*	1.19 (1.02, 1.38)*
≥7.2	1.43 (1.24, 1.65)***	1.37 (1.18, 1.60)***
Variation of DBP (%)		
<4.6	1.00	1.00
4.6–8.0	1.00 (0.86, 1.15)	1.00 (0.86, 1.16)
≥8.1	1.14 (0.99, 1.31)	1.18 (1.01, 1.37)*
Macroalbuminuria		
No	1.00	1.00
Yes	1.75 (1.24, 2.47)**	1.56 (1.10, 2.21)*

*Abbreviations*: HR, hazard ratio; CI, confidence intervals; DM, diabetes mellitus; FPG, fasting plasma glucose; SBP, systolic blood pressure; DBP, diastolic blood pressure.

*p < .05.

**p < .01.

***p < .001.

**TABLE 3:** Parameter estimates of regression coefficients and risk scores of predictors for new‐onset hypertension from the final multivariate Cox's proportional hazards model in derivation set

Risk factor	$\hat{\beta }$($\hat{SE}$)	*p*‐value	Risk score
***Socio‐demographic factors***			
Age (years)	0.02 (0.003)	<.001	0 to 10
Male	0.09 (0.06)	.13	1
***Lifestyle behaviors***			
Physical activity			
No	0.17 (0.06)	.006	2
Yes	Ref	Ref	0
Body mass index (kg/m^2^)			
<18.5	Ref	Ref	0
18.5‐24.9	0.68 (0.28)	.02	7
≥25.0	0.85 (0.28)	.003	8
***Diabetes related factors and biomarkers***			
Family history of hypertension			
No	Ref	Ref	0
Yes	0.25 (0.07)	<.001	2
Type of DM treatment			
Diet‐only	Ref	Ref	0
Any hypoglycemic drug	0.26 (0.12)	.03	3
Baseline systolic blood pressure (mm Hg)			
<120	Ref	Ref	0
120–129	0.39 (0.09)	<.001	4
130–139	0.72 (0.09)	<.001	7
Baseline diastolic blood pressure (mm Hg)			
<80	Ref	Ref	0
80–89	0.39 (0.07)	<.001	4
Variation of FPG (%)			
<10.9	Ref	Ref	0
10.9‐20.0	0.07 (0.08)	.37	1
≥20.1	0.15 (0.07)	.04	2
Variation of SBP (%)			
<4.0	Ref	Ref	0
4.0–7.1	0.17 (0.08)	.02	2
≥7.2	0.32 (0.08)	<.001	3
Variation of DBP (%)			
<8.1	Ref	Ref	0
≥8.1	0.16 (0.08)	.04	2
Macroalbuminuria			
No	Ref	Ref	0
Yes	0.44 (0.18)	.03	4

$\mathrm{Abbreviations}:\;\hat{\beta }$, Parameter estimate; $\hat{SE}$, standard error; DM, diabetes mellitus; FPG, fasting plasma glucose; SBP, systolic blood pressure; DBP, diastolic blood pressure.

**FIGURE 1:**
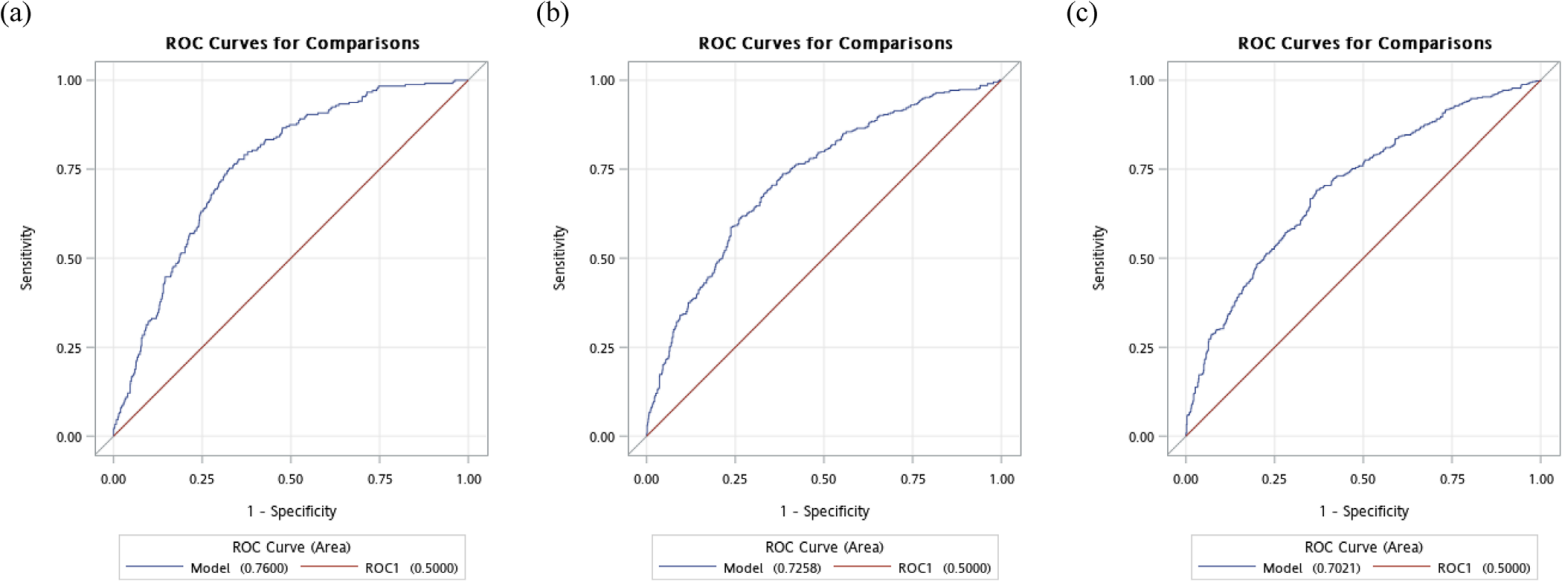
. Receiver operating characteristic curve (ROC) for (A) 1‐year (B) 3‐year (C) 5‐year hypertension risks in validation set

**FIGURE 2:**
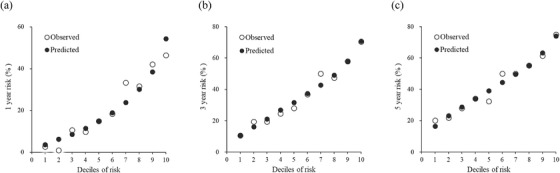
. Predicted versus observed risks of hypertension to deciles of (A) 1‐year (B) 3‐year and (C) 5‐year in validation set

**FIGURE 3:**
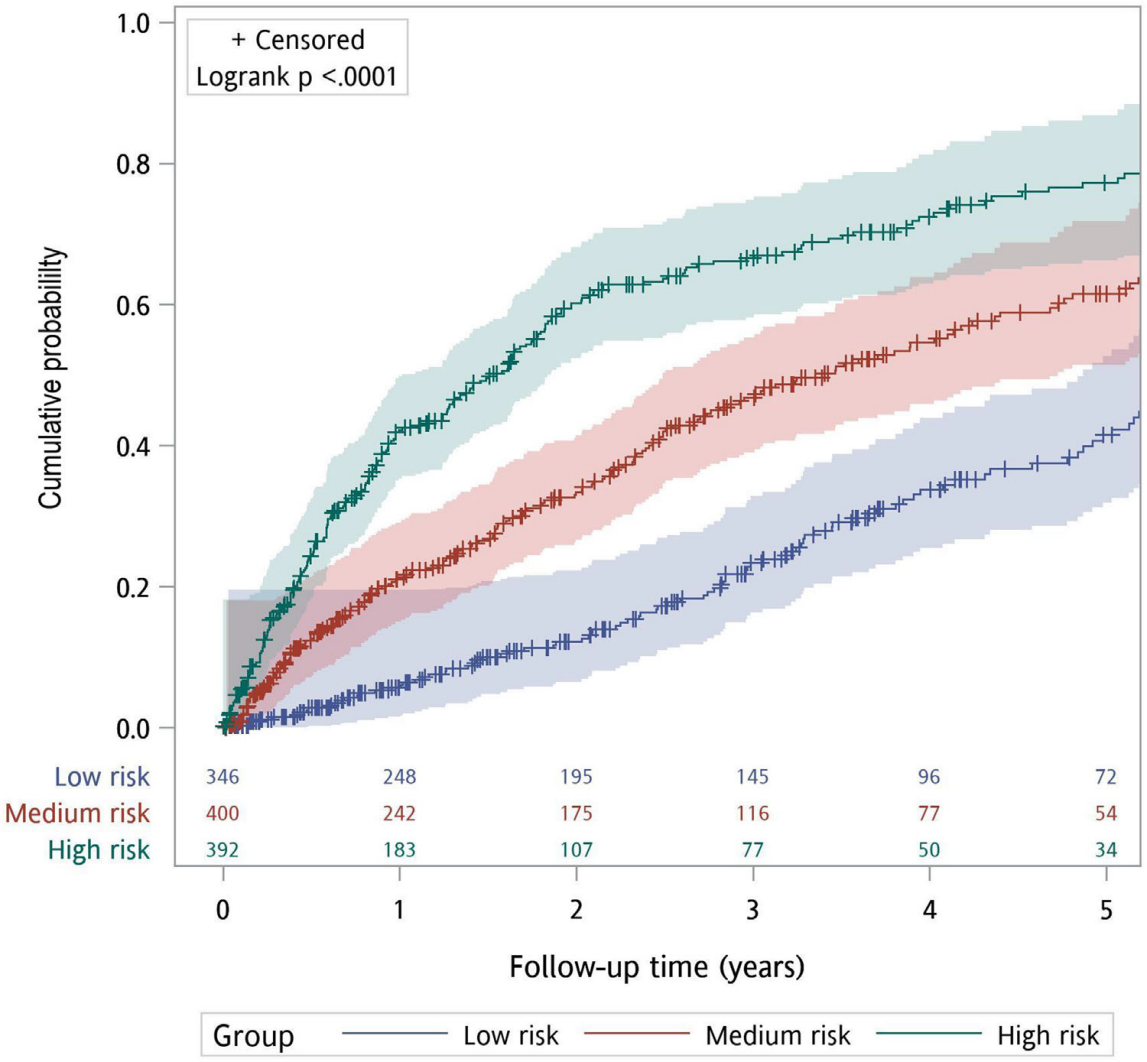
. Kaplan‐Meier estimates for cumulative hypertension incidence curves of the of the low, medium, and high‐risk groups in validation set

In the validation set, the scoring system exhibited good discriminative powers, as measured by the AUROC curve of 0.76 (95% CI: 0.73–0.79), 0.73 (95% CI: 0.70–0.76), and 0.70 (95% CI: 0.67–0.73), for predicting 1‐, 3‐, and 5‐year HTN incidence (Figure 1), respectively. The values of correct Harrell's C‐statistic were 0.70 (95% CI: 0.68–0.72) in the validation set, indicating the scoring system had good discriminatory capability. The calibration was assessed by 3‐, and 5‐year observed versus predicted plots (Figure 2) using the Hosmer–Lemeshow test (all *p >* .05), indicating the goodness of fit was acceptable. Subsequently, patients were divided into three groups based on approximate tertiles of the total scores and the proportions of low‐risk group (0–22 points), medium risk group (23–27 points), and high‐risk group (> 27 points) for predictive validation were 30.40%, 35.15%, and 34.45%, respectively. The Kaplan–Meier estimates for the cumulative HTN incidence curves of the three groups are shown in Figure 3 (log‐rank *p* < .001).

In this cohort, sensitivity, specificity, positive predictive value (PPV), and negative predictive value (NPV) were calculated at 40%, 50%, 70%, 80%, and 90%, risks, respectively, denoting a high risk (Table 4). Using a cutoff of > 22 points, the sensitivity, specificity, PPV, and NPV were 76.12%, 48.45%, 60.47%, and 66.21%, respectively. Thus, the proposed scoring system exhibited high values for specificity and PPV but low values for sensitivity and NPV.

**TABLE 4:** Sensitivity, specificity, PPV and NPV of the scoring system based on probability for HTN risk

						N (%)			
Probability for high risk	Risk scores	Sensitivity	Specificity	PPV	NPV	Patients classified as high risk	Patients classified as high risk who develop HTN	Patients classified as low risk	Patients classified as low risk who develop HTN
40%	19	89.07	26.58	55.68	70.13	2780 (81.38)	1548 (55.68)	636 (18.62)	190 (29.87)
50%	22	76.12	48.45	60.47	66.21	2188 (64.05)	1323 (60.47)	1228 (35.95)	415 (33.79)
60%	25	55.06	68.59	64.66	59.66	1480 (43.33)	957 (64.66)	1936 (56.67)	781 (40.34)
70%	27	39.47	80.51	67.72	56.22	1013 (29.65)	686 (67.72)	2403 (70.35)	1052 (43.78)
80%	30	19.62	92.73	73.65	52.69	463 (13.55)	341 (73.65)	2953 (86.45)	1397 (47.31)
90%	34	3.68	98.57	72.73	49.70	88 (2.58)	64 (72.73)	3328 (97.42)	1674 (50.30)

*Abbreviations*: PPV, Positive predictive value; NPV, Negative predictive value; HTN, hypertension.

In sensitivity analysis, persons with complete data were analyzed. In total, 2747 patients with type 2 diabetes were included in the final prediction model and yielded similar results with AUROC curve values of 0.74 (95% CI = 0.72–0.76), 0.70 (95% CI = 0.68–0.72), and 0.69 (95% CI = 0.67–0.71) for 1‐, 3‐, and 5‐year periods, respectively (Figure S2). Further, bootstrapping method was employed for internal validation with 1000 bootstrap samples. The intercept of a calibration curve was ‐0.0565, and the slope was 0.9823, showing a slight underestimation of HTN risk.

## 4 DISCUSSION

This study developed and validated a scoring system for the risk of HTN in patients with type 2 diabetes based on 13 predictors. To our knowledge, this research is the first attempt to establish a simple scoring system for the risk of HTN, with focus on patients with type 2 diabetes. The system had good discriminatory capability with a Harrell's C‐statistic of 0.70 (95% CI: 0.68–0.72) in the validation set. This scoring system considered risk factors that are generally accepted and available in clinical practice and are precisely measured to ensure its acceptability in clinical practice.

Diabetes is linked to impaired glucose tolerance or impaired fasting glucose through insulin resistance; concomitant islet beta‐cell injury may lead to insulin deficiency, which affects the utilization of glucose by skeletal muscles, adipose tissues, and hepatic cells.^36^ Insulin resistance increases tissue inflammation and reactive oxygen species production, thus resulting in endothelial dysfunction, inappropriate activation of the renin angiotensin aldosterone system, increased sympathetic nervous system activity, and abnormal sodium handling by the kidney.^37^ These responses have been implicated in the complex pathophysiology of HTN.^37^ Our study showed the significant association of FPG‐CV with HTN risk in the final predictive model. Similar results were also observed in Chien's study, which showed fasting glucose as a significant factor in the HTN prediction model.^23^

A review paper pointed out that early intervention by lifestyle modifications (weight loss, health dietary plan, reduction of dietary sodium intake, promotion of physical activity, and moderation of alcohol drinking) in persons with pre‐hypertensive condition can reduce BP or prevent HTN.^38^ The development of HTN prediction model for patients with type 2 diabetes will provide a rationale for the identification of high‐risk individuals and improve the efficiency of prevention and treatment strategies for HTN prevention in such individuals. The assigned scores for predictors in our scoring system provide information on HTN prevention for health professionals in clinical practice. General obesity, which can be prevented by lifestyle modification, and physical activity contributed to 10 points in our scoring system. Baseline SBP and DBP and variations in FPG, SBP, and DBP, accounting for 18 points, can also be modified by lifestyle or treatment intervention.

Most existing HTN risk prediction models achieve acceptable good discrimination with an AUC over 0.70,^5^, ^6^, ^8^, ^9^, ^10^, ^11^, ^12^, ^13^, ^14^, ^15^, ^16^, ^17^, ^18^, ^19^, ^20^, ^21^, ^22^, ^23^, ^24^, ^25^ and four of them consider diabetes status^11^, ^15^, ^24^ or fasting glucose.^23^ All prediction models have been developed using EHRs, primarily with consideration of general and patient populations and not of patients with diabetes. Thus, they cannot be used to consider diabetes‐related factors and may limit primary HTN prevention in patients with type 2 diabetes. Compared with USA HTN risk score,^24^ our scoring system showed an improvement in predicting risk, with improved prediction of events based on NRI (29.2%; 95% CI: 23.8%–34.5%, *p* < .001), where the probability for summation of likelihood for correctly reclassifying higher and incorrectly reclassifying lower among persons with events of 79.7% and probability for summation of likelihood for correctly reclassifying lower and incorrectly reclassifying higher among persons without events of –50.5%. That is, our scoring system is better than USA score for correct classifying those who will develop HTN, but it is worse for those who will not become hypertensives. The overall reclassification improvement favored the new score. The discrimination of risk based on IDI (5.0%; 95% CI: 3.4%–6.6%, *p* < .001) in Figure S3.

Our risk score system found new predictors such variation in FPG and BP in addition to traditional predictors. There is a barrier for the risk score to be used in clinical practice because of the need for calculating variability with several measurements when computing systems were not available. It can be overcome now because information systems are now common in most of clinical practice. The formula for calculating the variability can be integrated in the information systems and patients’ variability values can be provided for physicians. In addition, a smartphone app can facilitate its use in the settings for diabetes care.

### 4.1 Strengths and limitations

The strength of our study is the well‐defined patient group and pioneer scoring system for the early prediction of new‐onset HTN in patients with type 2 diabetes. Our scoring system considers traditional and novel predictors, including education level, physical activity, type of DM treatment, and glycemic and BP variabilities. The system showed a good discriminative capability in predicting 1‐, 3‐, and 5‐year risks. Our study also had potential limitations. First, glycemic and BP variabilities were calculated in persons with at least two measurement records during 1‐year period with 20% of missing values. To minimize the potential selection bias arising from missing data, we analyzed our data by using the MI approach for handling missing data. Using complete case analysis as sensitivity analysis and similar findings were obtained. Second, given the lack of external validation, we did not validate our system in an external or independent sample. External validation can provide evidence on the system's generalizability to various population. However, we performed internal validation with a bootstrapping method, and results showed that our system can be generalized to other populations with similar characteristics. Future research will be needed to examine external validation in independent datasets. Last, we didn't consider type of hypoglycemic drug because some categories such as insulin alone had very small sample size, resulting in the imprecision in estimating their effects. Because the effects of oral hypoglycemic drug use and insulin plus oral hypoglycemic use were similar, we collapsed all hypoglycemic drugs into a category.

## 5 CONCLUSIONS

We developed and validated a simple point‐based scoring system for HTN risk assessment using a hospital‐based EHR dataset of a managed care program. The scoring system showed good prediction capability, discriminatory power, and calibration. Our scoring system provides a valid and inexpensive tool to estimate medium‐term risks of new‐onset HTN in patients with type 2 diabetes and can help in preventing the progression of new‐onset HTN.

## CONFLICT OF INTEREST

There are no conflicts of interest.

## AUTHOR CONTRIBUTIONS

Tsai‐Chung Li, Cheng‐Chieh Lin, and Chia‐Ing Li were responsible for the conception and design of the work and writing manuscript. Chiu‐Shong Liu, Chih‐Hsueh Lin, and Mu‐Cyun Wang were responsible for data collection and data interpretation. Chia‐Ing Li and Shing‐Yu Yang were responsible for analysis. All authors read and approved the final manuscript.

## Supporting information

Supporting Information

## References

[1] Saeedi P , Petersohn I , Salpea P , et al. Global and regional diabetes prevalence estimates for 2019 and projections for 2030 and 2045: Results from the International Diabetes Federation Diabetes Atlas, 9th edition. Diabetes Research and Clinical Practice. 2019;157:107843. 10.1016/j.diabres.2019.107843. 31518657

[2] Bild D , Teutsch SM . The control of hypertension in persons with diabetes: a public health approach. Public Health Rep. 1987;102(5):522‐529. 3116583 PMC1477888

[3] Petrie JR , Guzik TJ , Touyz RM . Diabetes, hypertension, and cardiovascular disease: clinical insights and vascular mechanisms. Canad J Cardiol. 2018;34(5):575‐584. 29459239 10.1016/j.cjca.2017.12.005 PMC5953551

[4] Simonson DC . Etiology and prevalence of hypertension in diabetic patients. Diab Care. 1988;11(10):821‐827. 10.2337/diacare.11.10.821 3073072

[5] Wang B , Liu Y , Sun X , et‐al. Prediction model and assessment of probability of incident hypertension: the Rural Chinese Cohort Study. Journal of Human Hypertension. 2021;35(1):74–84. 10.1038/s41371-020-0314-8. 32107452

[6] Xu F , Zhu J , Sun N , et al. Development and validation of prediction models for hypertension risks in rural Chinese populations. J Glob Health. 2019;9(2):020601‐020601. 31788232 10.7189/jogh.09.020601 PMC6875679

[7] Li C , Sun D , Liu J , et al. A prediction model of essential hypertension based on genetic and environmental risk factors in Northern Han Chinese. Int J Med Sci. 2019;16(6):793‐799. 31337952 10.7150/ijms.33967 PMC6643104

[8] Kanegae H , Oikawa T , Suzuki K , et al. Developing and validating a new precise risk‐prediction model for new‐onset hypertension: the Jichi Genki hypertension prediction model (JG model). J Clin Hypertens. 2018;20(5):880‐890. 10.1111/jch.13270 PMC8031110 29604170

[9] Pei Z , Liu J , Liu M , et al. Risk‐predicting model for incident of essential hypertension based on environmental and genetic factors with support vector machine. Interdiscip Sci. 2018;10(1):126‐130. 29380342 10.1007/s12539-017-0271-2

[10] Du M , Yin S , Wang P , et al. Self‐reported hypertension in Northern China: a cross‐sectional study of a risk prediction model and age trends. BMC Health Services Res. 2018;18(1):475‐475. 10.1186/s12913-018-3279-3 PMC6006843 29921264

[11] Ye C , Fu T , Hao S , et al. Prediction of incident hypertension within the next year: prospective study using statewide electronic health records and machine learning. J Med Internet Res. 2018;20(1):e22. 29382633 10.2196/jmir.9268 PMC5811646

[12] Heo BM , Ryu KH . Prediction of prehypertenison and hypertension based on anthropometry, blood parameters, and spirometry. Int J Environ Res Public Health. 2018;15(11):2571. 10.3390/ijerph15112571 PMC6265931 30453592

[13] Sathish T , Kannan S , Sarma PS , Razum O , Thrift AG , Thankappan KR . A risk score to predict hypertension in primary care settings in rural India. Asia‐Pacific J Public Health. 2016;28(1):26S‐31S. 10.1177/1010539515604701 PMC4724234 26354334

[14] Chen Y , Wang C , Liu Y , et al. Incident hypertension and its prediction model in a prospective northern urban Han Chinese cohort study. J Hum Hypertens. 2016;30(12):794‐800. 27251078 10.1038/jhh.2016.23

[15] Niiranen TJ , Havulinna AS , Langén VL , et al. Prediction of blood pressure and blood pressure change with a genetic risk score. J Clin Hypertens. 2016;18(3):181‐186. 10.1111/jch.12702 PMC8032027 26435379

[16] Asgari S , Khalili D , Mehrabi Y , et al. Incidence and risk factors of isolated systolic and diastolic hypertension: a 10 year follow‐up of the Tehran Lipids and Glucose Study. Blood Press. 2016;25(3):177‐183. 26643588 10.3109/08037051.2015.1116221

[17] Otsuka T , Kachi Y , Takada H , et al. Development of a risk prediction model for incident hypertension in a working‐age Japanese male population. Hypertens Res. 2015;38(6):445. 26043932 10.1038/hr.2015.41

[18] Lu X , Huang J , Wang L , et al. Genetic predisposition to higher blood pressure increases risk of incident hypertension and cardiovascular diseases in Chinese. Hypertension. 2015;66(4):786‐792. 26283040 10.1161/HYPERTENSIONAHA.115.05961

[19] Choi Y‐H , Chowdhury R , Swaminathan B . Prediction of hypertension based on the genetic analysis of longitudinal phenotypes: a comparison of different modeling approaches for the binary trait of hypertension. BMC Proceedings. 2014;8(S1). 10.1186/1753-6561-8-s1-s78. PMC4143688 25519406

[20] Li G , Liu J , Wang W , et al. [Prediction models for the 15 years risk of new‐onset hypertension in Chinese people aged from 35 to 64 years old]. Zhonghua Nei Ke Za Zhi. 2014;53(4):265‐268. 24857297

[21] Carson AP , Lewis CE , Jacobs DR , et al. Evaluating the Framingham hypertension risk prediction model in young adults: the Coronary Artery Risk Development in Young Adults (CARDIA) study. Hypertension. 2013;62(6):1015‐1020. 24041951 10.1161/HYPERTENSIONAHA.113.01539 PMC4019674

[22] Völzke H , Fung G , Ittermann T , et al. A new, accurate predictive model for incident hypertension. J Hypertens. 2013;31(11):2142‐2150. 24077244 10.1097/HJH.0b013e328364a16d

[23] Chien K‐L , Hsu H‐C , Su T‐C , et al. Prediction models for the risk of new‐onset hypertension in ethnic Chinese in Taiwan. J Human Hypertens. 2011;25(5):294‐303. 20613783 10.1038/jhh.2010.63

[24] Kshirsagar AV , Chiu Ya‐L , Bomback AS , et al. A hypertension risk score for middle‐aged and older adults. J Clin Hypertens. 2010;12(10):800‐808. 10.1111/j.1751-7176.2010.00343.x PMC3683833 21029343

[25] Tsimihodimos V , Gonzalez‐Villalpando C , Meigs JB , et al. Hypertension and diabetes mellitus: coprediction and time trajectories. Hypertension. 2018;71(3):422‐428. 29335249 10.1161/HYPERTENSIONAHA.117.10546 PMC5877818

[26] Mariye T , Girmay A , Tasew H , et al. Determinants of hypertension among diabetic patients in Public Hospitals of the Central Zone. Pan Afr Med J. 2019;33:100. 31489078 10.11604/pamj.2019.33.100.17094 PMC6711702

[27] Ohara M , Nagaike H , Yamamoto T , Hayashi T , Fukui T , Hirano T . Effects of Glucose and Blood Pressure Variability on Oxidative Stress in Type 2 Diabetes with Hypertension. Diabetes. 2018;67(Supplement 1):411–P. 10.2337/db18-411-p.

[28] Di Flaviani A , Picconi F , Di Stefano P , et al. Impact of glycemic and blood pressure variability on surrogate measures of cardiovascular outcomes in type 2 diabetic patients. Diab Care. 2011;34(7):1605‐1609. 10.2337/dc11-0034 PMC3120198 21610126

[29] Levey AS , Stevens LA , Schmid CH , et al. A new equation to estimate glomerular filtration rate. Ann Intern Med. 2009;150(9):604‐612. 19414839 10.7326/0003-4819-150-9-200905050-00006 PMC2763564

[30] Unger T , Borghi C , Charchar F , et al. International society of hypertension global hypertension practice guidelines. Hypertension. 2020;75(6):1334‐1357. 32370572 10.1161/HYPERTENSIONAHA.120.15026

[31] Kilpatrick ES , Rigby AS , Atkin SL . A1C variability and the risk of microvascular complications in type 1 diabetes: data from the diabetes control and complications trial. Diab Care. 2008;31(11):2198‐2202. 10.2337/dc08-0864 PMC2571045 18650371

[32] Sullivan LM , Massaro JM , D'agostino RB . Presentation of multivariate data for clinical use: the Framingham Study risk score functions. Stat Med. 2004;23(10):1631‐1660. 15122742 10.1002/sim.1742

[33] Steyerberg E , Clinical Prediction Models–A Practical Approach to Development, Validation, and Updating. New York: Springer, 2009.

[34] Ramirez LA , Sullivan JC . Sex differences in hypertension. Am J Hypertens. 2018;31(12):1247‐1254. 30299518 10.1093/ajh/hpy148 PMC6233684

[35] Guo X , Zou L , Zhang X , et al. Prehypertension: a meta‐analysis of the epidemiology, risk factors, and predictors of progression. Texas Heart Inst J. 2011;38(6):643‐652. PMC3233334 22199424

[36] Stumvoll M , Goldstein BJ , Van Haeften TW . Type 2 diabetes: principles of pathogenesis and therapy. Lancet. 2005;365(9467):1333‐1346. 15823385 10.1016/S0140-6736(05)61032-X

[37] Manrique C , Lastra G , Gardner M , et al. The renin angiotensin aldosterone system in hypertension: roles of insulin resistance and oxidative stress. Med Clin North America. 2009;93(3):569‐582. 10.1016/j.mcna.2009.02.014 PMC2828938 19427492

[38] Zhang W , Li N . Prevalence, Risk Factors, and Management of Prehypertension. International Journal of Hypertension. 2011;2011:1–6. 10.4061/2011/605359. PMC3205676 22121474

