# Supplementary material for: A risk scoring system to predict the risk of new‐onset hypertension among patients with type 2 diabetes
Source: J Clin Hypertens (Greenwich). 2021 Jul 12;23(8):1570–80. doi: 10.1111/jch.14322 (PMC8678759; doi:10.1111/jch.14322)
Supplement: Supplementary file 1 — Supporting Information [file JCH-23-1570-s001.docx]

**Supplement A: Steps for variable selection in multivariate Cox’s proportional hazard model and building up prediction model**

The model-building process included four steps for picking out independent variables to produce the “best” model. First, we executed a univariate analysis on each independent variable. Next, we chose an independent variable with a p-value<0.25 in univariate analysis as a candidate for our multivariable analysis [Bendel RB, 1997]. Then, we built a multivariable model with candidate variables and simultaneously checked the possibility of collinearity. Only independent variables with p < 0.05 can be kept in the final multivariate model. If there existed high collinearity within a subset of variables, their significance and estimated their regression coefficients were compared with their values in univariate analysis. The variable that explained an outcome best was kept in the multivariate Cox model. Lastly, the assumption of Cox’s proportional hazard model and potential interactions for all variables in the multivariate model was evaluated.

The steps for building up the prediction model are as follows:

Step 1: To estimate the parameters of the multivariable Cox’s proportional hazards model.

Step 2: To classify the risk or protective factors into categories and to decide the reference value W_i_.

Step 3: To assign a score for each category for deciding the referent risk factor profile, where a base category of each risk or protective factor is assigned 0 score.

Step 4: To decide the distance from the base category to each category in regression units.

Step 5: To set the constant B, the number of regression units reflecting 1 point in the final point system. B was defined as five-fold the regression coefficient of age.

Step 6: To calculate the number of points for each category of each risk or protective factor, where Point_ij_ =(𝑊_𝑖𝑗_ −𝑊_𝑖𝑅𝐸𝐹_)/B.

Step 7: To determine the predictive risks for all possible total scores.

The predictive risks for HTN were estimated by the equation: $\hat{p}=1-P_{0}{(t)}^{exp(\sum\beta_{i}\times X_{i}-\beta_{i}\times\bar{X}_{i})}$, where 𝑃_0_ is the baseline HTN-free probability, 𝛽_𝑖_ is the regression coefficient for *X_i_* , and the $\bar{X}_{i}$ is the mean level of *X_i._*

Bendel RB, Afifi AA: Comparison of stopping rules in forward stepwise regression. *J Am Stat Assoc* 1977, 72(357):46-53.

**Supplemental Table S1.** 1-, 3-, 5-year estimated risks for hypertension of each possible sum of points.

| Point total | Predicted risk of hypertension | | |
| --- | --- | --- | --- |
|  | 1-year risk, % | 3-year risk, % | 5-year risk, % |
| 0 | 2.23% | 5.19% | 7.77% |
| 1 | 2.46% | 5.73% | 8.55% |
| 2 | 2.72% | 6.31% | 9.41% |
| 3 | 3.00% | 6.96% | 10.36% |
| 4 | 3.31% | 7.66% | 11.38% |
| 5 | 3.66% | 8.44% | 12.51% |
| 6 | 4.03% | 9.28% | 13.74% |
| 7 | 4.45% | 10.21% | 15.07% |
| 8 | 4.91% | 11.23% | 16.53% |
| 9 | 5.41% | 12.34% | 18.10% |
| 10 | 5.97% | 13.55% | 19.81% |
| 11 | 6.58% | 14.87% | 21.66% |
| 12 | 7.25% | 16.31% | 23.66% |
| 13 | 7.98% | 17.87% | 25.81% |
| 14 | 8.79% | 19.56% | 28.11% |
| 15 | 9.67% | 21.39% | 30.57% |
| 16 | 10.63% | 23.36% | 33.20% |
| 17 | 11.69% | 25.49% | 35.99% |
| 18 | 12.84% | 27.77% | 38.94% |
| 19 | 14.10% | 30.21% | 42.04% |
| 20 | 15.47% | 32.81% | 45.28% |
| 21 | 16.95% | 35.58% | 48.66% |
| 22 | 18.57% | 38.50% | 52.15% |
| 23 | 20.32% | 41.58% | 55.74% |
| 24 | 22.21% | 44.81% | 59.39% |
| 25 | 24.24% | 48.17% | 63.08% |
| 26 | 26.43% | 51.64% | 66.77% |
| 27 | 28.78% | 55.22% | 70.42% |
| 28 | 31.29% | 58.86% | 73.99% |
| 29 | 33.96% | 62.55% | 77.44% |
| 30 | 36.79% | 66.24% | 80.73% |
| 31 | 39.78% | 69.90% | 83.80% |
| 32 | 42.93% | 73.48% | 86.64% |
| 33 | 46.21% | 76.95% | 89.20% |
| 34 | 49.62% | 80.27% | 91.46% |
| 35 | 53.14% | 83.37% | 93.42% |
| 36 | 56.75% | 86.25% | 95.06% |
| 37 | 60.42% | 88.85% | 96.41% |
| 38 | 64.11% | 91.15% | 97.47% |
| 39 | 67.79% | 93.15% | 98.29% |
| 40 | 71.43% | 94.84% | 98.88% |
| 41 | 74.97% | 96.23% | 99.31% |
| 42 | 78.38% | 97.33% | 99.59% |
| 43 | 81.61% | 98.18% | 99.77% |
| 44 | 84.62% | 98.81% | 99.88% |
| 45 | 87.38% | 99.25% | 99.94% |
| 46 | 89.86% | 99.56% | 99.97% |
| 47 | 92.04% | 99.75% | 99.99% |
| 48 | 93.91% | 99.87% | 100.00% |

18,373 adults enrolled in the National Diabetes Care Management Program

Excluding

With type 1 diabetes, gestational diabetes (n=448)

Age<30 or >85 (n=655)

History of hypertension or less than one-year of follow-up (n=13,603)

3,667 subjects were eligible

Excluding

Without baseline information (n=251)

Without variations in blood pressure and glucose level (n=669)

2,747 participants were included for compete-case analysis as sensitivity analysis

Training Set

n=2,278

Validation Set

n=1,138

Random allocation by 2:1 ratio

3,416 participants were included for main analysis

Excluding

Without baseline information (n=251)

Imputing

Without variations in blood pressure and glucose level (n=669)

**Supplemental Figure S1.** Flowchart for recruitment procedures of the hypertension prediction model.

| (a)  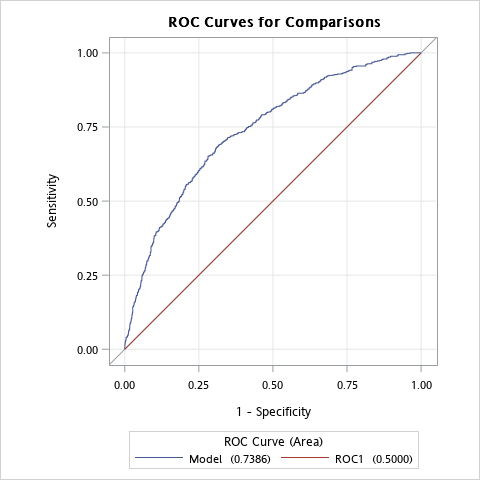 | (b)  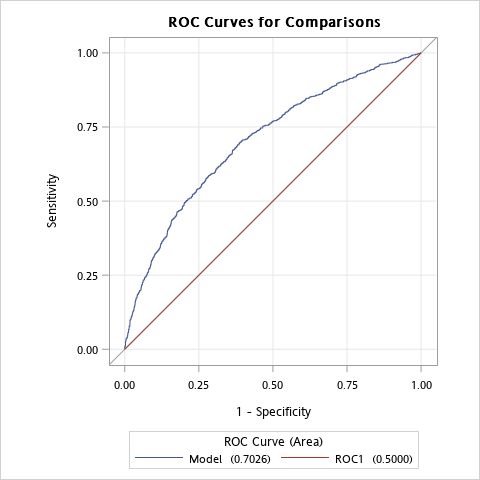 | (c)  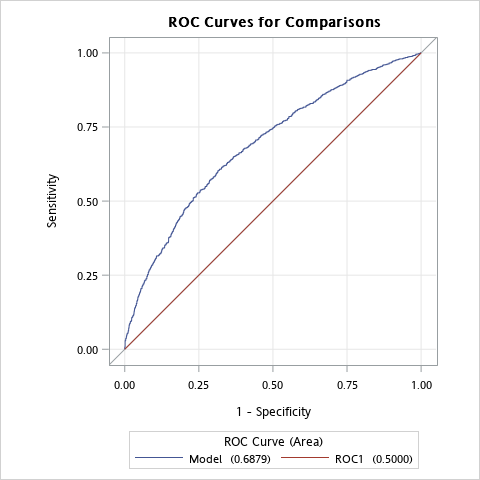 |
| --- | --- | --- |

**Supplemental Figure S2**. The areas under the receiver operating characteristics curve (AUROC) for (a) 1-year (b) 3-year (c) 5-year hypertension risk in sensitivity analysis (n=2,747)

| **(a)Events** | | | | | |
| --- | --- | --- | --- | --- | --- |
|  |  | Risk in TW score | | |  |
|  |  | <42% | 42-59% | ≥59% | Total |
| Risk in USA score | <42% | 78  18.8% | 114  27.5% | 151  36.5% | 343  82.8% |
|  | 42-59% | 0  0.0% | 6  1.5% | 65  15.7% | 71  17.2% |
|  | ≥59% | 0  0.0% | 0  0.0% | 0  0.0% | 0  0.0% |
|  | Total | 78  18.8% | 120  29.0% | 216  52.2% | 414  100% |

| **(b) Non-Events** | | | | | |
| --- | --- | --- | --- | --- | --- |
|  |  | Risk in TW score | | |  |
|  |  | <42% | 42-59% | ≥59% | Total |
| Risk in USA score | <42% | 344  47.5% | 192  26.5% | 134  18.5% | 670  92.5% |
|  | 42-59% | 2  0.3% | 10  1.4% | 42  5.8% | 54  7.5% |
|  | ≥59% | 0  0.0% | 0  0.0% | 0  0.0% | 0  0.0% |
|  | Total | 346  47.8% | 202  27.9% | 176  24.3% | 724  100% |

**Supplemental Figure S3.** The net reclassification improvement (NRI) table comparing the Taiwan (TW) and USA scores for individuals who (a) did or (b) did not have hypertension events for 3-year in validation set
